# Single-Cell Profiling Reveals Transcriptional Signatures and Cell-Cell Crosstalk in Anti-PLA2R Positive Idiopathic Membranous Nephropathy Patients

**DOI:** 10.3389/fimmu.2021.683330

**Published:** 2021-05-31

**Authors:** Jie Xu, Chanjuan Shen, Wei Lin, Ting Meng, Joshua D. Ooi, Peter J. Eggenhuizen, Rong Tang, Gong Xiao, Peng Jin, Xiang Ding, Yangshuo Tang, Weisheng Peng, Wannian Nie, Xiang Ao, Xiangcheng Xiao, Yong Zhong, Qiaoling Zhou

**Affiliations:** ^1^ Department of Nephrology, Xiangya Hospital, Central South University, Changsha, China; ^2^ Department of Hematology, The Affiliated Zhuzhou Hospital Xiangya Medical College, Central South University, Zhuzhou, China; ^3^ Department of Pathology, Xiangya Hospital, Central South University, Changsha, China; ^4^ Centre for Inflammatory Diseases, Monash University Department of Medicine, Monash Medical Centre, Clayton, VIC, Australia; ^5^ Department of Organ Transplantation, Xiangya Hospital, Central South University, Changsha, China; ^6^ Department of Ultrasonography, Xiangya Hospital, Central South University, Changsha, China

**Keywords:** single-cell RNA sequence, idiopathic membranous nephropathy, kidney, immune response, inflammation

## Abstract

Idiopathic membranous nephropathy (IMN) is an organ-specific autoimmune disease of the kidney glomerulus. It may gradually progress to end-stage renal disease (ESRD) characterized by increased proteinuria, which leads to serious consequences. Although substantial advances have been made in the understanding of the molecular bases of IMN in the last 10 years, certain questions remain largely unanswered. To define the transcriptomic landscape at single-cell resolution, we analyzed kidney samples from 6 patients with anti-PLA2R positive IMN and 2 healthy control subjects using single-cell RNA sequencing. We then identified distinct cell clusters through unsupervised clustering analysis of kidney specimens. Identification of the differentially expressed genes (DEGs) and enrichment analysis as well as the interaction between cells were also performed. Based on transcriptional expression patterns, we identified all previously described cell types in the kidney. The DEGs in most kidney parenchymal cells were primarily enriched in genes involved in the regulation of inflammation and immune response including IL-17 signaling, TNF signaling, NOD-like receptor signaling, and MAPK signaling. Moreover, cell-cell crosstalk highlighted the extensive communication of mesangial cells, which infers great importance in IMN. IMN with massive proteinuria displayed elevated expression of genes participating in inflammatory signaling pathways that may be involved in the pathogenesis of the progression of IMN. Overall, we applied single-cell RNA sequencing to IMN to uncover intercellular interactions, elucidate key pathways underlying the pathogenesis, and identify novel therapeutic targets of anti-PLA2R positive IMN.

## Introduction

Idiopathic membranous nephropathy (IMN) is a common cause of nephrotic syndrome (NS) in adults with a peak occurrence of 50-60 years old (1). It is characterized by subepithelial immune deposits, complement-mediated proteinuria, and risk of kidney failure. The prevalence of IMN is increasing worldwide, particularly in elderly patients, and has been reported in 20.0–36.8% of adult-onset NS cases (2–5). The clinical outcome of patients is quite variable, with spontaneous remission reported in up to one-third of cases and progression to end-stage renal disease (ESRD) in a similar number (6–8).

IMN is a noninflammatory autoimmune disease of the kidney glomerulus (9, 10). In the last 10 years, substantial advances have been made in the understanding of the molecular bases of IMN, with the identification of several antigens [neutral endopeptidase, phospholipase A2 receptor (PLA2R), thrombospondin domain-containing 7A (THSD7A)] and the characterization of antibody-binding domains of these auto-antigens. 50% to 80% of the patients will test positive for an anti-PLA2R antibody with any of the available tests depending on the state of disease activity (11, 12). These ground-breaking findings already have a major impact on diagnosis and therapy monitoring. Besides, several risk alleles, such as HLA-DQ, HLA-DR, and PLA2R1 have been identified as risk factors of IMN (13, 14). The pathogenesis of IMN induced by podocyte *in situ* antibody and the following complement activation pathways have been revealed to some extent (9, 15, 16). However, the reason for the heterogeneity of patients as well as the variety of clinical outcomes remains elusive. Furthermore, a comprehensive analysis of the cell types and molecular pathways involved in IMN is lacking.

Single-cell RNA-sequencing (scRNA-seq) is a transcriptomic technology that measures the expression of up to thousands of genes in thousands of single cells simultaneously. It offers an opportunity to comprehensively describe human kidney disease at a cellular level and plays a crucial role in identifying cell subtypes and illustrating molecular differences (17). This technique has been applied to several complex kidney diseases including kidney cell carcinoma, diabetic nephropathy, lupus nephritis, and acute kidney injury (18–21). Here we applied scRNA-seq to kidney biopsies of patients with IMN to identify gene expression at the single-cell level, elucidate cells involved in the progression of IMN, and uncover intercellular interactions.

## Materials and Methods

### Ethical Approval and Consent

The Medical Ethics Committee of the Xiangya Hospital of Central South University for Human Studies approved the study (ID: 201711836). The implementations were in concordance with the International Ethical Guidelines for Research Involving Human Subjects as stated in the Declaration of Helsinki. Informed written consent was obtained from participants or their legal guardians.

### Tissue Procurement

Kidney specimens were obtained from the department of nephrology in Xiangya Hospital, Central South University. We conducted a kidney biopsy with 18-gauge core needles in the nephrotic syndrome subjects paralleling with positive serum anti-PLA2R antibody. Healthy adult kidney tissues were collected by biopsy of living donor kidneys from two transplant donors. Healthy kidney tissue was collected after removal from the donor and before implantation into the recipient. Kidney tissues were cleaned with sterile phosphate buffered saline (PBS) after collection.

### Kidney Sample Processing and Single-Cell Dissociation

Fresh kidney tissue specimens were stored in GEXSCOPE Tissue Preservation Solution (Singleron Biotechnologies) at 2-8°C immediately. The specimens were washed with Hanks’ Balanced Salt Solution (HBSS) three times and then minced into 1-2 mm pieces before dissociation. Single-cell suspensions were obtained by digestion with 2ml GEXSCOPE Tissue Dissociation Solution (Singleron Biotechnologies) with continuous agitation at 37°C for 15min. The samples were subsequently filtered through 40-μm sterile cell strainers (Corning) to separate cells from cell debris and other impurities, after which they were centrifuged at 300 x g for 5 minutes at 4°C and cell pellets were resuspended into 1ml PBS (HyClone). Next, 2ml GEXSCOPE Red Blood Cell Lysis Buffer (Singleron Biotechnologies) was added into the cell suspension and incubated at 25°C for 10 minutes to remove red blood cells. The cells were then centrifuged at 300 x g for 5 min and resuspended in cold PBS for downstream analyses. Quantification of cell yields was performed by TC20 automated cell counter (Bio-Rad) with trypan blue exclusion, once the cell viability exceeded 70%, subsequent sample processing could be performed.

### Library Preparation and Preprocessing of scRNA-Seq Data

PBS was added to the single-cell suspension to adjust the concentration to 1×10^5^ cells/mL. A single-cell suspension was then loaded onto the microfluidic chip. The single-cell RNA-seq libraries were prepared according to the manufacturer’s protocol using the Singleron GEXSCOPE Single Cell RNA-seq Library Kit (Singleron Biotechnologies), which included cell lysis, mRNA trapping, labeling cells (barcode) and mRNA (UMI), reverse transcription mRNA into cDNA and amplification, and finally fragment cDNA. Samples were then sequenced by Hiseq X10 (Illumina, San Diego, CA, USA) with 150bp paired-end reads. Raw reads were processed to generate gene expression profiles using an internal pipeline. Adapters and poly-A tails were trimmed (fastp V1) before aligning read two to GRCh38 with ensemble version 92 gene annotation (fastp 2.5.3a and featureCounts 1.6.2). Reads with the same cell barcode, UMI, and gene were grouped to calculate the number of UMIs per gene per cell.

### Cell Type Classification and Marker Genes Analysis

The Seurat program (http://satijalab.org/seurat/, R package, v.3.0.1) was applied for the analysis of RNA-Sequencing data including cell type identification and clustering analysis. By default, we used the SNN (shared nearest neighbor) model of the Seurat program package for clustering analyses and displayed the distribution status of cells by dimension reduction operation (PCA, tSNE, UMAP). Next, Wilcox (Wilcoxon rank-sum test) was used to analyze the difference of each cluster and the result of the marker gene obtained by “Wilcox” (Likelihood-ratio test) using the FindAllMarkers function in Seurat combined the differential gene list above identified the marker gene of each cluster. The selected marker genes were expressed in over 10% of the cells per cluster and the average log (Fold Change) was more than 0.25. The heatmap was completed by the top20 marker genes of each cell cluster. Sub-clustering analysis of endothelial cells and myeloid immune cells was performed by the SubsetData function of the Seurat.

### Differentially Expressed Genes Identified Between Groups

Differentially expressed genes (DEGs) of each kidney cell cluster were identified by comparing the transcriptional profile of IMN and healthy donors. We performed “Wilcox”(Likelihood-ratio test)by the FindAllMarkers function in Seurat to determine the DEGs of each cluster between the two groups. DEGs were defined by a gene with an average log (Fold Change) exceeded 0.25 and P-value smaller than 0.05.

### Enrichment and Cell Interaction Analysis

Gene Ontology (GO) function enrichment analysis was performed on the gene set using the clusterProfiler software to find biological processes or molecular functions that are significantly associated with the genes specifically expressed. Similarly, Kyoto Encyclopedia of Genes and Genomes (KEGG) enrichment analysis was carried out to get significantly related pathways by the clusterProfiler software. We also conducted a ligand-receptor interaction analysis of cell-cell cross-talk by CellphoneDB.

## Results

Extensive clinical, laboratory, and pathologic evaluation were required to separate primary from secondary MN and help determine the underlying etiology (22). All patients were reviewed for potential secondary causes of MN such as hepatitis serology, antinuclear antibodies, anti–double-stranded DNA antibodies, anti−Smith antibodies, complements, chest radiographs, age-appropriate cancer screening, medication history, and monoclonal gammopathy evaluation. All biopsies were reviewed for histologic features suggestive of secondary MN, such as full house immunofluorescence, vascular or tubular basement membrane deposits on immunofluorescence, and mesangial and endothelial proliferation, as well as for the presence of endothelial tubuloreticular inclusions or mesangial deposits on electron microscopy (23). Patients with potential secondary causes were excluded from further analysis. Kidney biopsy samples were obtained from 6 patients with IMN and 2 healthy controls. Patients with IMN were positive for serum anti-PLA2R antibody and evidenced by the diffuse formation of subepithelial “spikes”, or heterogeneous thickening of the glomerular basement membrane by light microscopy, as well as subepithelial electron-dense deposits and diffuse fusion of podocyte foot processes by electron microscopy (**Supplemental Figure 1**). Serum anti-PLA2R antibody levels ranged from 26.28 to 816.47 RU/ml and the pathologic stage varied from II-IV. Also, patient ages ranged from 34 to 65 years and one of them was female. The proteinuria of these six IMN patients varied from 1.18 to 11.35 g per 24h. IMN patients were divided into massive proteinuria group and non-massive proteinuria group with the critical value of 3.5g urinary protein excretion per day. Four of the six IMN patients had more than 3.5g proteinuria per 24h in our study. Albumin (ALB) and triglyceride (TG) were 22.22 ± 1.00 g/L and 1.96 ± 0.42 mmol/l respectively. The estimated glomerular filtration rate (eGFR) of IMN patients ranged from 61.91 to 107 mL/min/1.73m^2^, and the mean serum creatinine (Scr) was 1.08 ± 0.11 mg/dL. Serum C3 concentration of all subjects was normal whereas serum IgG levels in four IMN patients were below the lower limit of normal (**Supplemental Table 1**). At the time of biopsy, all IMN patients have not received medications other than RAAS inhibitors. As for healthy control, one kidney donor was a 47-year-old male, and his creatinine was 0.68 mg/dl, eGFR was 113.81 mL/min/1.73m^2^. Another kidney donor was a 50-year-old male, and his creatinine was 0.94 mg/dl, eGFR was 94.16 mL/min/1.73m^2^. The two donors neither have diseases history including hypertension and diabetes nor any medications prescribed before.

### Cell Lineage in the Kidney Identified by scRNA-Seq

**Figure 1:**
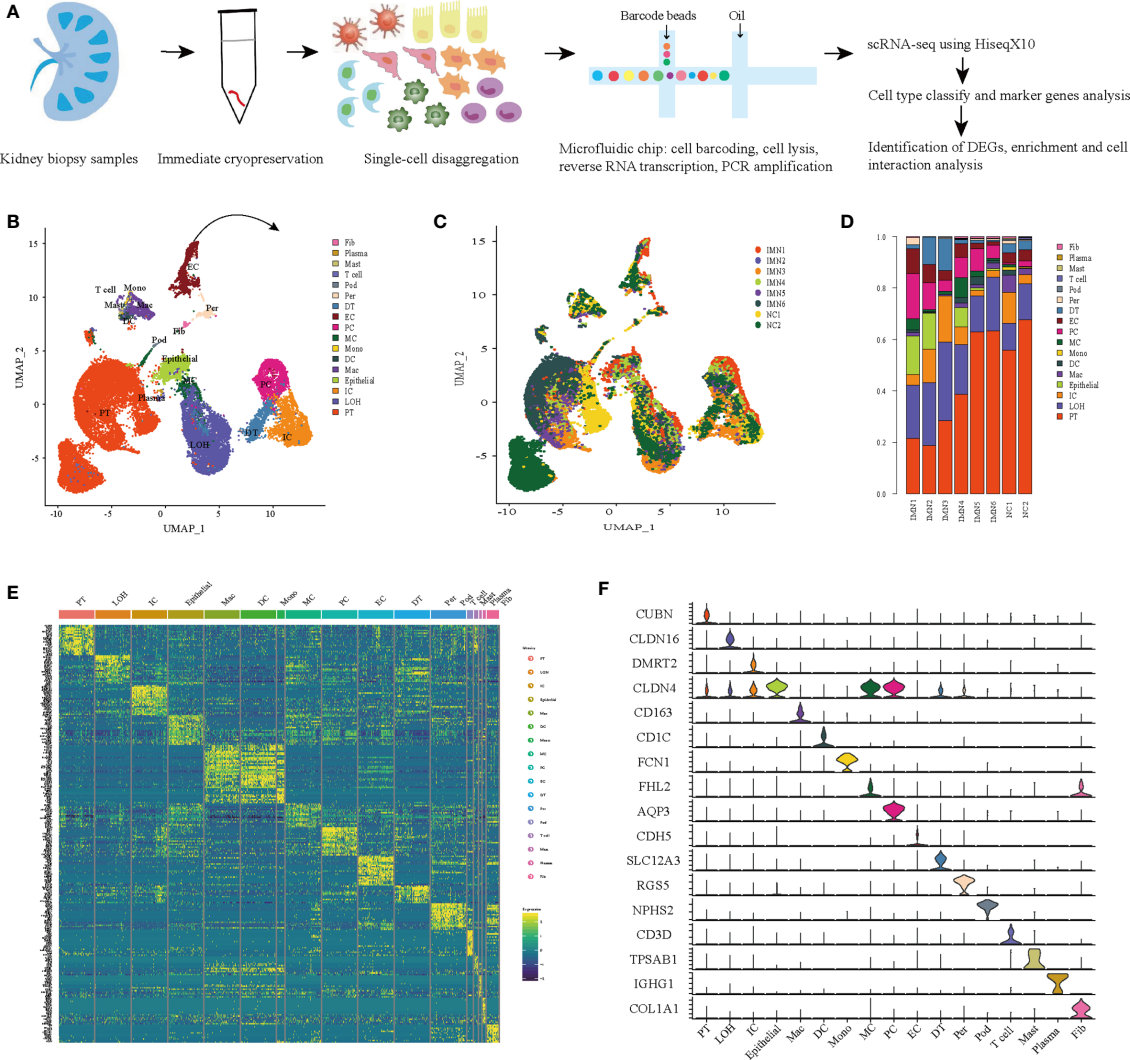
Cell lineage analysis by comprehensive single-cell RNA-sequencing in anti-PLA2R positive IMN and control subjects. **(A)** Schematic of the scRNA-seq pipeline. Kidney samples from patients with IMN (n=6) or healthy control subjects (n=2) were collected at the time of clinically indicated renal biopsy or live kidney donation, respectively. Kidney biopsies were enzymatically disaggregated into single-cell suspensions and loaded onto a microfluidic device for cell barcoding, cell lysis, reverse RNA transcription, and then scRNA-seq as well as various other analyses. **(B)** Seventeen distinct cell clusters were visualized by UMAP plotting, with each cell color-coded for its associated subtypes. The color of the cells represents group origin. **(C)** UMAP plot of cell clusters from different subjects of IMN patients and control. The color of cells reflected the individual origin. **(D)** Bar plots of the percent contribution of cell clusters in kidneys from different subjects. Blocks represented different subjects, and block height was in proportion to the number of cells. **(E)** Heatmap of the top 20 most differentially expressed genes in each cluster to identify mutually exclusive gene sets, which were then used to determine the cell lineage of each cluster. Each column represented a cell cluster, and each row corresponded to a marker gene for the individual cluster. Transcript abundance ranges from low (purple) to high (yellow). **(F)** Violin plot of selected marker genes that identified the clusters generated by UMAP plotting. It was colored by different cell subtypes. PT, proximal tubule cells; LOH, loop of Henle cells; PC, principal cells; IC, intercalated cells; DT, distal tubule cells; EC, endothelial cells; Pod, podocytes; MC, mesangial cell; DC, dendritic cells; Mac, macrophages; Mono, monocytes; Fib, fibroblasts; Per, pericyte.

We first catalogued kidney cell types of all eight subjects in an unbiased manner using droplet-based single-cell RNA sequencing. After data pre-processing and stringent quality control, transcriptomic data were obtained from 30313 cells (**Figure 1A**). The number of cells for each sample varied from 2047 to 8879 and cell viability ranged from 71% to 98% (**Supplemental Table 2**). Eleven kidney subsets and six immune subsets consisting of as few as 21 cells to as many as 13792 cells per cluster, were isolated by a graph-based clustering approach and labeled according to lineage-specific markers following batch correction (**Figure 1B**). The cell distribution from eight different kidney subjects was visualized by uniform manifold approximation and projection (UMAP) (**Figure 1C**). To define the character of each cell cluster, differential expression analysis was carried out to identify mutually exclusive sets of genes and therefore established markers of particular cell types. Enrichment of different cell clusters was calculated for each subject respectively (**Figure 1D**). The top 20 most differentially expressed markers in each cluster were shown in **Figure 1E**, and the selected cell lineage-specific marker gene was displayed in **Figure 1F**. For example, endothelial cells uniquely expressed *CDH5* and *KDR*, podocytes uniquely expressed *NPHS2* and *PODXL*, whereas mesangial cells expressed *FN1* and *FHL2*. Pericyte was labeled by *RGS5* and *ACTA2*. In addition, proximal tubule cells distinctly expressed *CUBN*, distal tubule cells distinctly expressed *SLC12A3*, whereas the loop of Henle cells uniquely expressed *CLDN16*. *DMRT2* and *SLC4A1* were defined as a cell-type specific marker for intercalated cells while *AQP3* were expressed specifically in principal cells. Fibroblasts expressed many genes encoding extracellular matrix proteins including *COL1A1* and *DCN*. The comprehensive and detailed cell-lineage-specific marker genes of different kidney cells were displayed in **Table 1**.

**Table 1:** Cell-lineage-specific marker genes of different kidney cells.

Cell Type	Abbreviation	Marker genes
Proximal tubule cells	PT	CUBN, SLC13A1, LRP2, ALDOB
Mesangial cells	MC	FHL2, FN1, MYL9, CTGF
Podocytes	Pod	NPHS2, PODXL, PTPRO
Loop of Henle cells	LOH	UMOD, SLC12A1, CLDN16
Distal tubule cells	DT	CALB1, SLC12A3
Intercalated cells	IC	SLC4A1, ATP6V0D2, FOXI1, DMRT2
Principal cells	PC	AQP2, AQP3, GATA3
Epithelial cells	Epi	EPCAM, KRT8, CLDN4
Endothelial cells	EC	CDH5, PECAM1, KDR, CLDN5
Fibroblasts	Fib	COL1A1, DCN, LUM
Pericytes	Per	RGS5, ACTA2, MCAM, PDGFRB
T cells	T cell	CD3D, TRBC1, CD3E
Plasma cells	Plasma	IGHG1, JCHAIN, MZB1
Mast cells	Mast	TPSAB1, TPSB2, CPA3
Macrophages	Mac	MRC1, CD68, CD163, C1QA, IL1B
Monocytes	Mono	LYZ, CD14, VCAN, FCN1
Dendritic cells	DC	CD1C, FCER1A, CLEC10A, IRF8

### Identification of DEGs and Enrichment Analysis in the Kidney Cells of Anti-PLA2R Positive IMN Subjects

**Figure 2:**
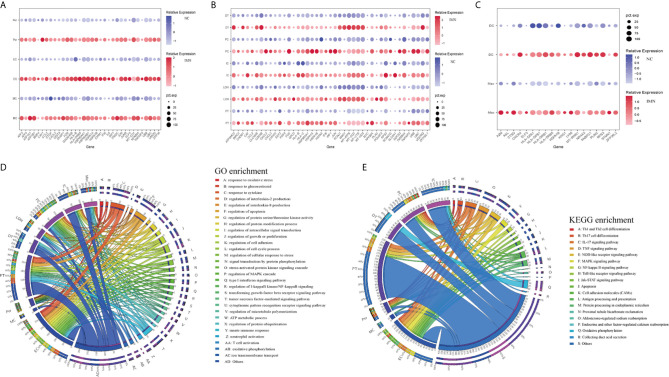
DEGs and enrichment analysis in the kidney cells of anti-PLA2R positive IMN and control subjects. **(A)** Representative DEGs in mesangial cells, endothelial cells, and pericytes comparing the IMN patients to healthy donor control. pct.exp: percentage of cells expressing gene. **(B)** Representative DEGs in proximal tubule cells, distal tubule cells, loop of Henle cells, principal cells, and intercalated cells between IMN patients and control. **(C)** Representative DEGs in immune cells between IMN patients and control. **(D, E)** GO and KEGG enrichment shows the biological processes or signal pathways involved in different kidney cells, respectively. The left side of the circle represents different cell types, while the right side represents different biological processes or signaling pathways. The inner-circle represents gene numbers involved in cells or biological processes and signaling pathways, whereas the outer-circle represents the proportion of each cell type in biological processes and signaling pathways or the proportion of biological processes and signaling pathways in kidney cells.

To explore gene expression changes in kidney parenchymal cells, we performed differential expression analysis of transcriptomes between IMN patients and healthy donors. DEGs of kidney cells from the glomerulus and tubules were provided in **Datasets 1** and **2**, respectively. We defined representative DEGs in glomerular intrinsic cells (**Figure 2A**) as well as tubular intrinsic cells (**Figure 2B**) by comparing the transcriptional profile between IMN and control subjects.

Mesangial cells (MCs) of IMN highly expressed *IFI6* and *ATF3*. *IFI6*, a gene induced by interferon, which is associated with the regulation of apoptosis and type I interferon signaling pathway (24), was upregulated in MCs of IMN. Mesangial cells of IMN highly expressed ATF3, which was demonstrated to promote sublytic C5b-9-induced MCs apoptosis through up-regulation of *GADD45A* and *KLF6* gene expression (25). Besides, *KLF4*, *UBB, UBC*, and *DUSP1* were upregulated in endothelial cells of IMN. Endothelial *KLF4* mediated the protective effect of statins through regulating the expression of cell adhesion molecules and concomitant recruitment of inflammatory cells (26), which was implied by GO analysis in our study. *UBB* and *UBC*, both the important component of the ubiquitin pathway, were elevated in endothelial cells of IMN. They may play an essential role in the regulation of cell cycle, signal transduction as well as programmed cell death (27). Endothelial cells expressed *DUSP1*, a kind of two-way specific threonine/tyrosine phosphatase that regulates the mitogen-activated protein kinase (MAPK) signaling pathway by dephosphorylation of threonine/serine and tyrosine residues on its target (28) and regulates cell proliferation and cell growth cycle. Pericytes, a multifunctional cell-type of the kidney, highly expressed *FOS* that might play a role in the proliferation of pericytes and respond to PDGF-BB stimulation by phosphorylating both the PDGF receptor and the MAP kinase ERK-1/2 (29). Pericytes expressed CCL2 which might play a role in pericyte activation, proliferation, and differentiation into myofibroblasts during progressive kidney injury (30). GO enrichment analysis showed that DEGs were enriched in the regulation of apoptosis and type I interferon signaling pathway in mesangial cells, the regulation of programmed cell death, and various cytokine-mediated signaling pathways in endothelial cells and the regulation of protein modification in pericytes (**Figure 2D**), while KEGG enrichment analysis revealed that DEGs were mainly associated with the IL-17 signaling, TNF signaling, NOD-like receptor signaling and MAPK signaling pathway in endothelial cells as well as pericytes (**Figure 2E**).

DEGs upregulated in proximal tubules cells (PT) such a*s HSPA1A*, *TNFAIP3*, *KNG1*, and *TMSB4X*, were enriched in the regulation of cell proliferation, adhesion, programmed cell death, and response to cytokines in GO enrichment analysis (**Figure 2D**), whereas *NFKBIA*, *CXCL2*, *JUN*, *BIRC3* DEGs were enriched by KEGG enrichment analysis and participate in IL-17 signaling, TNF signaling, NOD-like receptor signaling, and NF-kappa B signaling (**Figure 2E**). Comparison of the DEGs in the distal tubule cells (DT) displayed enrichment of genes involved in oxidative phosphorylation, ATP metabolic process, and cation transmembrane transport (**Figure 2D**). The loop of Henle cells (LOH) of IMN had increased expression of *PLAU*, *KNG1*, *EEF2*, and *CAT*, which contribute to neutrophil-mediated immunity, exocytosis, and adherens junction (**Figure 2D**). A total of 2160 and 2194 cells were present in the principal cells (PC) and intercalated cells (IC), respectively. As illustrated in **Figures 2D, E**, DEGs of PCs between IMN and control subjects were enriched in IL-17 signaling, TNF signaling, NOD-like receptor signaling, and pattern recognition receptor signaling, of which *NFKBIA*, *JUN*, *CCL2*, *UBC* were involved. In addition to the genes responsible for acid secretion, including *SLC4A1*, *CLCNKB*, and *ATP6V1F*, DEGs of ICs were also enriched involving in adherens junction, oxidative phosphorylation, and organic compound catabolic process (**Figures 2D, E**).

Six clusters of leukocytes in the kidney of IMN were identified according to cell-specific differential genes, which were composed of dendritic cells (DC), macrophages, monocytes, mast cells, plasma cells, and T cells. The DEGs dataset of these leukocytes is displayed in **Figure 2C** and **Dataset 3**, except for monocytes, which were unable to be analyzed due to insufficient cell numbers. Also, the deficiency of meaningful differential genes in plasma cells, T cells, and mast cells was probably owing to the technical limits of isolating insufficient corresponding cells, since previous studies have confirmed their contributions in IMN (31–33). Macrophages highly expressed genes responsible for the regulation of leukocyte activation (*ZFP36L2*, *AXL*, and *RPS3*), inflammatory response (*KNG1*, *ELF3*, *CXCR4*, and *LY86*), and the antigen processing and presentation as well as immune response-regulating signaling pathway (*HLA-DPA1*, *HLA-DPB1*, and *HLA-DRB1*) (**Figures 2D, E**). Detailed information on the expression of the genes discussed above was provided in **Dataset 4**.

### Cell-Cell Crosstalk in Anti-PLA2R Positive IMN Through Ligand-Receptor Interactions

**Figure 3:**
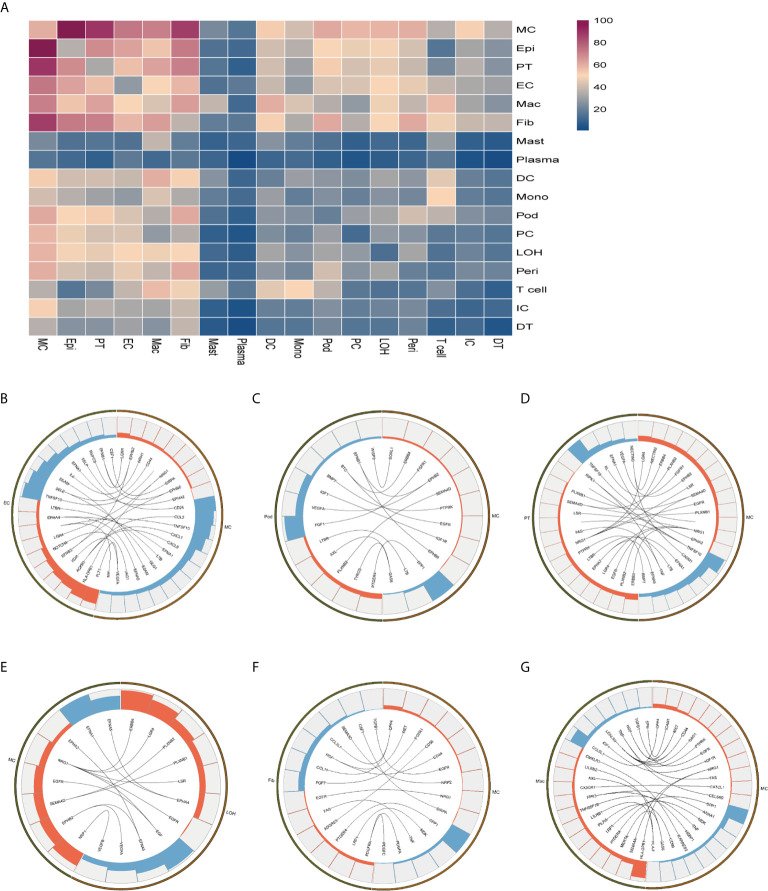
Possible ligand-receptor interactions between different cell types in the kidney of anti-PLA2R positive IMN patients. **(A)** Ligand-receptor signaling pathways between cell clusters in the kidney. Cell-cell crosstalk frequency ranges from low (blue) to high (purple). **(B)** Representative ligand-receptor interactions between mesangial cells and endothelial cells. **(C)** Representative ligand-receptor interactions between mesangial cells and podocytes. **(D)** Representative ligand-receptor interactions between mesangial cells and proximal tubule cells. **(E)** Representative ligand-receptor interactions between mesangial cells and loop of Henle cells. **(F)** Representative ligand-receptor interactions between mesangial cells and fibroblasts. **(G)** Representative ligand-receptor interactions between mesangial cells and macrophages. Lines represented interrelations between the mesangial cells and other cells. Lines between the ligand and conjunct receptors were shown. Only IMN patients (n=6) were analyzed.

**Figure 4:**
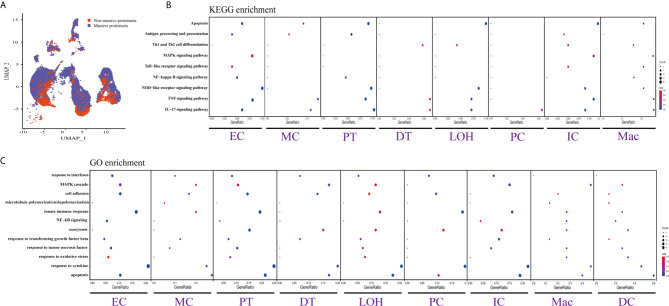
DEGs involved in biological process and intercellular signaling in the kidney from anti-PLA2R positive IMN patients with massive proteinuria compared to non-massive proteinuria. **(A)** UMAP plot of cell clusters between massive proteinuria and non-massive proteinuria. The color of cells reflected different groups. **(B, C)** KEGG and GO enrichment analysis showed that upregulated DEGs were mostly involved in apoptosis, cell adhesion, and regulation of inflammation and the immune response in massive proteinuria patients, compared with non-massive proteinuria subjects.

To explore the interactions and signaling network of different cell subsets in IMN, we performed ligand-receptor analysis. **Figure 3A** displayed the potential interactions of receptors and ligands in different cell types of kidneys. *CXCL1*, *CXCL8*, or *CCL2* expressed by mesangial cells interacted with *ACKR1* in endothelial cells (**Figure 3B**), which may participate in neutrophil/macrophage infiltration and inflammation response (34). FGF1 expressed by podocytes might ameliorate chronic kidney disease *via* PI3K/AKT mediated suppression of oxidative stress and inflammation (35) under the expression of FGFR1 in mesangial cells (**Figure 3C**). Moreover, PT expressed PTPRK, an important cell-cell adhesion regulator realized by reversible phosphorylation of protein tyrosine residues (36). We found it may interact with BMP7 from mesangial cells (**Figure 3D**). EGF, expressed by the loop of Henle cells, interacts with EGFR or NGR1 in mesangial cells (**Figure 3E**), which probably plays a role in cell proliferation (37). Mesangial cells highly expressed SPP1 and become interaction pairs with PTGER4 from fibroblasts (**Figure 3F**), possibly involved in the activation of T cells. In addition to PTGER4 in fibroblasts, we found SPP1 in mesangial cells might interact with PTGER4 in macrophages (**Figure 3G**). The remaining information of cell-cell crosstalk was all displayed in **Figure 4**.

### DEGs and Enrichment Analysis From Anti-PLA2R Positive IMN Patients Between Massive Proteinuria Group and Non-Massive Proteinuria Group

We displayed cell distribution between the massive proteinuria group and the non-massive proteinuria group from IMN patients by a graph-based clustering approach (**Figure 4A**). The grouping was based on whether proteinuria reached the scope of nephrotic syndrome, which is 3.5g per 24h. We then compared DEGs of proteinuria between the massive proteinuria group and the non-massive proteinuria group from IMN patients. The more specific information of DEGs from various kidney cells in two groups of IMN was shown in **Dataset 5**. ECs, MCs, PTs, LOHs, DTs, as well as ICs in IMN patients with massive proteinuria had elevated expression of *KLF6* that might participate in the glomerular mesangial cell proliferation, ECM accumulation, and proteinuria secretion (38). All the intrinsic kidney cells except for podocytes, pericytes, fibroblasts, and epithelial cells in IMN patients with massive proteinuria highly expressed SOCS3, a cytokine-inducible protein that might contribute to the regulation of receptor signaling in immune complex glomerulonephritis, in parallel with proteinuria and kidney lesions (39). Besides, all tubular cells in IMN patients with massive proteinuria had a significant expression of MMP7, which was secreted as a soluble protein from the tubules to the glomeruli and mediated the impairment of slit diaphragm integrity, leading to podocyte dysfunction and increased proteinuria (40). This suggests MMP-7 might be the key mediator of tubular-to-glomerular crosstalk that promotes proteinuria and CKD progression.

As exhibited in **Figures 4B, C**, DEGs between the massive proteinuria group and the non-massive proteinuria group from IMN patients were enriched in several common biological processes. For example, the regulation of apoptosis was enriched in MCs, LOHs, PTs, DTs, PCs, and ICs, whereas adherens junctions were enriched in PTs, LOHs, and DTs. Besides, the overexpressed genes in MCs, PTs, DTs, PCs, and ICs were mainly enriched in the response to cytokines, while most of the kidney parenchymal cells were enriched in the regulation of inflammation and immune response including TNF signaling, NOD-like receptor signaling, MAPK signaling as well as IL-17 signaling pathways.

## Discussion

Our understanding of the pathogenesis of IMN is limited by an incomplete molecular characterization of the cell types in the kidney and interaction between the cells. Given the organ-specific immunological characteristics of IMN, we performed unbiased single-cell RNA sequencing for the first time and identified all previously described cell types in the kidney. The DEGs in most kidney parenchymal cells were primarily enriched in the regulation of inflammation and immune response including IL-17 signaling, TNF signaling, NOD-like receptor signaling as well as MAPK signaling. Besides, the cell-cell crosstalk highlighted the extensive communication of mesangial cells, which infers great importance in IMN.

We provided abundant information of cell-type-specific gene expression and distinct signaling pathways by analysis of DEGs as well as enrichment. Glomerular cells including MCs, ECs, and pericytes, primarily participated in the regulation of programmed cell death, inflammatory process, and immune regulation, whereas tubular cells are mainly involved in adherens junction, oxidative phosphorylation as well as regulation of inflammation and immunity. *HSPA1A*, *ATF3*, *IFI6*, and *ITM2C*, which were significantly expressed in all glomerular cells and enriched in regulation of apoptosis, have not yet been implicated in IMN pathogenesis. Also, DEGs of the ECs, pericytes, PTs, and PCs, are related to the inflammatory process and immunity. Particularly, Th17 cells are key players in kidney autoimmunity by mediating fundamental inflammatory cascades and thereby may be of vital importance in IMN (41, 42). Previously, studies reported that IL-17 has several direct effects on kidney parenchymal cells facilitating leukocyte transmigration, promoting interaction with T-cells, and impacting kidney integrity (43, 44). These effects are inherent to the pathophysiological cascade in kidney autoimmunity (45). For example, a study demonstrated that tubular epithelial cells showed signs of disrupted cell-cell junctional integrity and loss of E-cadherin expression after exposure to IL-17 (44, 46), which is consistent with our scRNA-seq findings in kidney interstitial cells. We also observed extensive enrichment of NOD-like receptors (NLRs) signaling in glomerular as well as tubular cells. NLRs are recently identified intracellular PRRs that are essential to innate immune responses and tissue homeostasis. Emerging evidence suggested a potential role of NLRs in kidney disease (47, 48). Expression of Nod1, Nod2, or RICK induces NF-κB activation (49). In addition to NF-κB, Nod1 and Nod2 mediate the activation of JNK and p38 in response to microbial ligands (50, 51), which are expected to participate in the transcriptional activation of proinflammatory genes. However, to the best of our knowledge, none of the genes or pathways discussed above were explored in IMN patients. Thus, further studies are needed to validate our results and provide novel insights into the pathogenesis of human IMN.

The cell-cell crosstalk through ligand-receptor interactions was reported to show considerable importance in anti-PLA2R positive IMN pathogenesis (52). We focused on the regulation of inflammation and immunity between different cells, especially mesangial cells for their extensive communication with other cells in our study. A study demonstrated that lysophosphatidylcholine might stimulate EGF receptor transactivation and downstream MAP kinase signaling resulting in mesangial hypercellularity (53), while EGF similarly stimulated MAPK (ERK1/2) in HK-2 cells and consequently mediate cell proliferation (37), which might imply crosstalk between glomerular and tubular cells. Macrophages highly expressed *PTGER4*, which has been shown to drive the differentiation of Th1 cells and proliferation of Th17 cells (54). While SPP1, also known as osteopontin (OPN*)*, was upregulated in mesangial cells. Coincidentally, a study demonstrated that OPN functionally activates DCs and induces their differentiation toward a Th1-polarizing phenotype (55), which implies the possibility of communications between macrophages and mesangial cells. Furthermore, there are some hints that *CXCL1*, *CXCL8*, or *CCL2* expressed by mesangial cells interacted with *ACKR1* in endothelial cells. ACKR1, better known as Duffy antigen receptor for chemokines (DARC or Duffy), is usually thought to regulate the innate and adaptive immune response by acting as a chemokine reservoir or scavenger, and it seems to be a negative regulator of inflammation and immunological stimuli through combination with chemokines including CXCL1, CXCL8 and CCL2 (56). Chaudhuri et al. also demonstrated that vascular endothelial cells may induce Duffy protein to regulate leukocytes and chemokine trafficking (57). The communication of cells in the kidney is highly dynamic but more efforts are essential to elucidate the precisely controlled process.

As a crucial clinical indicator in various kidney diseases, proteinuria drew our attention as a matter of course. The enrichment analysis revealed that most of the intrinsic kidney cells were involved in inflammatory pathways in IMN patients with massive proteinuria, suggesting substantial functions in the disease setting. MAPK signaling was associated with proteinuria. A recent study reported that p38 MAPK mediated secretory phospholipase A2 group IB-induced autophagy in podocytes and promoted podocyte injury *via* activation of the mTOR/ULK1^ser757^ signaling pathway, which consequently lead to proteinuria (58). ERK and p38 pathways also mediated activation of calcium-independent phospholipase A2γ, which plays an important role in complement C5b-9-induced glomerular epithelial cell (GEC) injury and proteinuria (59). TNF signaling was also involved in IMN progression with proteinuria. Active membranous glomerulonephritis leads to not only proteinuria but also increased urinary TNF excretion (60). However, inhibition of TNF signaling might attenuate kidney immune cell infiltration in experimental membranous nephropathy (61). Also, circulating tumor necrosis factor receptors (cTNFRs) were suggested to predict renal progression in patients with IMN accompanied by nephrotic syndrome (62).

However, there were several limitations to our study. First, the number of patients in this study was small, which may inevitably lead to individual differences. To reduce this difference, additional specimens are needed to verify the results of our study. Second, as the podocytes are particularly important and even the core for membranous nephropathy (63–65), podocytes were rarely detected in this study is one major limitation. Currently, it is still a major challenge for the capture of rare cells, such as podocytes in the application of single-cell transcriptomics technologies to kidney diseases. The podocytes have not been successfully annotated in several previous single-cell sequencing studies focusing on kidney diseases because of the very small proportion of podocytes after digestion of needle kidney biopsy tissue (66–69). In our study, we clearly captured and annotated the population of podocytes. There were 27 and 23 podocytes captured in 6 patients with IMN and 2 healthy donors, respectively. However, we only found one differentially expressed gene due to the small number of podocytes captured in the disease group as well as healthy control group. A further increase in throughput of the next generation of single-cell sequencing techniques or extracting the glomerular from kidney tissue before dissociation into single cells may prove efficient to capture enough podocytes for subsequent analysis (70). Third, as no other kidney disease control group was included in this study, the changes of DEGs are not specific for anti-PLA2R positive IMN and the changes may be generic to being proteinuria or glomerular inflammation.

Overall, scRNA-seq served as a feasible and valuable technique performed in IMN patients. We demonstrated cell-specific transcriptional profiles in the kidney, anti-PLA2R positive IMN-associated novel genes, signaling pathways involved, and potential pathogenesis concerning ligand-receptor interactions. A better understanding of the molecular mechanism in IMN will provide yet unexplored opportunities to develop new therapies for kidney diseases. The results discovered in our study will be further validated using tissue staining, functional studies *in vitro* using cell lines or primary human cells, and animal models of IMN.

## Data Availability Statement

The original contributions presented in the study are publicly available. This data can be found here: https://www.ncbi.nlm.nih.gov/, GSE171458.

## Author Contributions

YZ, QZ, CS, JO, and PE conceived and supervised the project. JX and WL performed all biopsy dissociations and single-cell experiments. XD, PJ, TM, WN, XA, GX, and YT assisted with patient consent and sample acquisition of IMN biopsies and assisted with patient consent and sample acquisition of live kidney donor tissue. Renal biopsy histology was evaluated by WL. Analysis was performed by YZ, JX, XA, and RT. JX, YZ, and QZ prepared and wrote the manuscript. JO and PE revised the paper. All authors contributed to the article and approved the submitted version.

## Conference Presentation

Parts of the present study have been accepted as a Mini-Oral at the 58th ERA-EDTA Congress, which will be organized from June 5 to 8, 2021.

## Funding

This work was funded by the National Key R&D Program of China (2020YFC2005000 to XX), the National Natural Science Foundation of China (81800641 to TM), the Key Research and Development Program of Hunan province (2018WK2060 to XX and 2020WK2008 to YZ), the science and technology innovation Program of Hunan Province (2020RC5002 to JO), the Natural Science Foundation of Hunan Province (2020JJ6109 to CS and 2019JJ40515 to WN), Chinese Society of Nephrology (18020010780 to YZ).

## Conflict of Interest

The authors declare that the research was conducted in the absence of any commercial or financial relationships that could be construed as a potential conflict of interest.
