# Supplementary material for: Single-Cell Profiling Reveals Transcriptional Signatures and Cell-Cell Crosstalk in Anti-PLA2R Positive Idiopathic Membranous Nephropathy Patients
Source: Front Immunol. 2021 May 31;12:683330. doi: 10.3389/fimmu.2021.683330 (PMC8202011; doi:10.3389/fimmu.2021.683330)
Supplement: Supplementary file 1 [file DataSheet_1.docx]

***Supplementary Materials***

**Supplemental Table 1. Demographic and biochemical characteristics of patients with IMN**

**Supplemental Table 2. Number of cells and viability for each sample**

**Supplemental Figure 1. Pathology characteristics of IMN patients**

**Dataset 1. DEGs in glomerulus cells from IMN and control subjects**

Abbreviations were as follows: pct.1, the percentage of cells where the gene is detected in the IMN patients; pct.2, the percentage of cells where the gene is detected in the control subjects; avg logFC, log fold-change of the average expression between the two groups, positive values indicate that the gene is more highly expressed in the first group.

**Dataset 2. DEGs in tubular cells from IMN and control subjects**

**Dataset 3. DEGs in immune cells from IMN and control subjects**

**Dataset 4. Detailed information of selected genes in kidney**

**Dataset 5. DEGs in different cell clusters of the kidney from IMN patients with** **massive proteinuria compared to non-massive proteinuria**

**Supplemental Table 1. Demographic and biochemical characteristics of patients with IMN**

|  | IMN1 | IMN2 | IMN3 | IMN4 | IMN5 | IMN6 |
| --- | --- | --- | --- | --- | --- | --- |
| Age, Gender | 64, M | 61, M | 47, F | 65, M | 52, M | 34, M |
| BP (mmHg) | 120/64 | 180/120 | 129/92 | 135/89 | 128/78 | 125/77 |
| Diabetes mellitus | No | No | No | No | No | No |
| HGB (g/L) | 141 | 145 | 124 | 137 | 126 | 118 |
| TG (mmol/L) | 2.2 | 1.45 | 2.56 | 1.53 | 1.7 | 2.31 |
| TC (mmol/L) | 7.73 | 6.06 | 5.75 | 5.49 | 7.59 | 9.56 |
| Serum ALB (g/L) | 22.1 | 24.1 | 21.6 | 21.9 | 22.7 | 20.9 |
| Scr (mg/dL) | 1.17 | 0.97 | 0.63 | 1.14 | 1.09 | 1.46 |
| Serum UA (umol/L) | 373.4 | 456.8 | 303.8 | 375.3 | 495 | 439.9 |
| Serum C3(mg/L) | 789 | 1020 | 868 | 915 | 893 | 1020 |
| Serum IgG (mg/L) | 3.37 | 9.57 | 5.36 | 12.1 | 6.18 | 6.15 |
| Serum anti-PLA2R antibody (RU/mL) | 65.05 | 77.15 | 26.28 | 816.47 | 48.19 | 75.11 |
| eGFR  mL/min/1.73m^2^ | 65.75 | 84.32 | 107 | 66.93 | 77.58 | 61.91 |
| Proteinuria (g/24h) | 10.75 | 3.54 | 1.18 | 8.02 | 2.34 | 11.35 |
| Urinary protein / creatinine (g/g) | 9.72 | 1.68 | 2.01 | 6.53 | 1.68 | 8.92 |
| Pathologic stage of IMN | III | II | II-III | II-III | III | III-IV |

ALB, albumin; Scr, serum creatinine; UA, uric acid; TG, triglyceride; TC, total cholesterol; HGB, hemoglobin; eGFR, estimated glomerular filtration rate; NS, Nephrotic syndrome.

**Supplemental Table 2. Number of cells and viability for each sample**

| Sample No. | Control 1 | Control 2 | IMN 1 | IMN 2 | IMN 3 | IMN4 | IMN5 | IMN6 |
| --- | --- | --- | --- | --- | --- | --- | --- | --- |
| Total cells | 4322 | 6084 | 8879 | 2047 | 7106 | 4114 | 2930 | 5573 |
| Viability (%) | 92.50% | 91.10% | 71.00% | 98.00% | 80.00% | 80.90% | 77.50% | 84.00% |

**Supplemental Figure 1. Pathology characteristics of IMN patients**

(A) Light microscopy shows heterogeneous thickening of the glomerular basement membrane accompanied by a mild proliferation of mesangial cells in IMN patients (HE X400). (B) Periodic acid-silver methe-namine (PASM) staining shows obvious deposition of fuchsinophilic substances along the basement membrane and the formation of “spikes” with the silver stain. The internal vacuolizations in part of the GBM are apparent (X400). (C) Positive anti-PLA2R staining along the capillary wall by immunofluorescence (X200). (D) Positive IgG4 deposit along the capillary wall by immunofluorescence (X200). (E and F) Electron microscopy shows that electron-dense substance was deposited under the glomerular visceral epithelial cells and extensive podocyte fusion (E X5000 & F X7000).

**
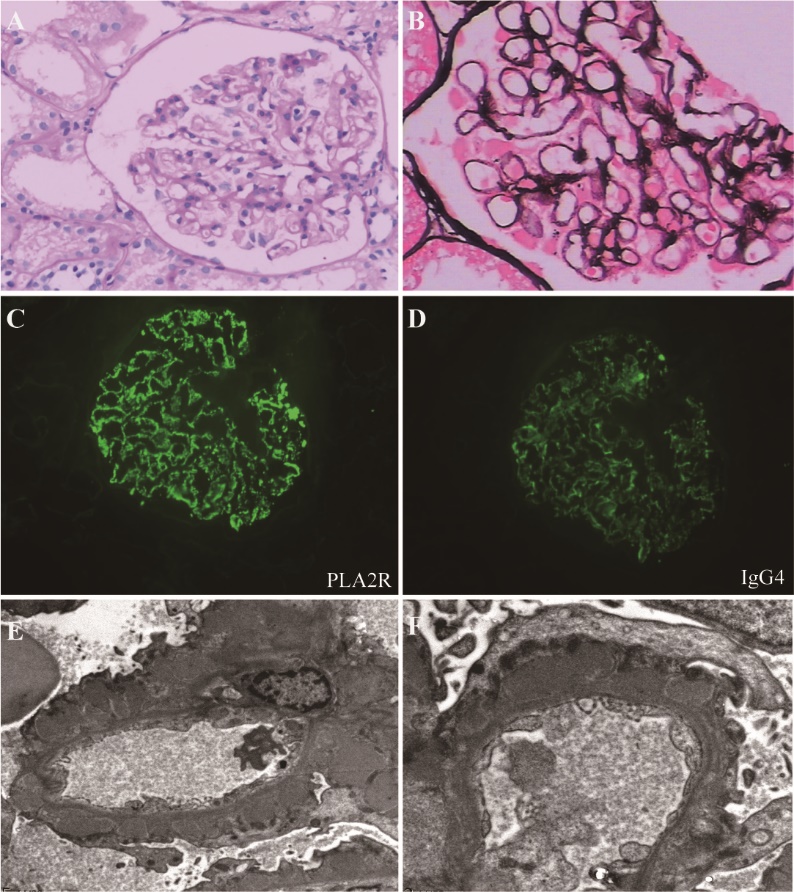
**

**Dataset 1-5** were available at the supplementary materials as Excel.
